# Impact of Cytomegalovirus (CMV) on an Academic Pediatric Infectious Diseases Outpatient Clinic Referral Population, 2005–2020: Will the Advent of Universal Congenital CMV (cCMV) Screening Change Clinical Practice Referral Patterns?

**DOI:** 10.3390/ijns10010014

**Published:** 2024-02-15

**Authors:** Katelyn J. Rypka, Mark R. Schleiss

**Affiliations:** 1 University of Minnesota Medical School, 420 Delaware Street SE, Minneapolis, MN 55455, USA; rypka008@umn.edu; 2 Division of Infectious Diseases, Department of Pediatrics, University of Minnesota Medical School, 2001 6th Street SE, Minneapolis, MN 55455, USA

**Keywords:** congenital cytomegalovirus (cCMV), sensorineural hearing loss (SNHL), targeted screening, universal newborn screening, pediatric infectious diseases (PID) clinic, ganciclovir, valganciclovir, newborn hearing screen (NHS)

## Abstract

Cytomegalovirus (CMV) infections exert a substantial impact on the practice of pediatric infectious diseases. Although most infections in children are minimally symptomatic, several populations are at risk for CMV-associated disease, including immunosuppressed children, children with HIV infection, and, most significantly, children with congenital CMV (cCMV) infection. In spite of the ubiquitous nature of CMV infection, few studies have quantified the impact of CMV-associated care in a pediatric outpatient clinic setting. We evaluated the impact of CMV on clinical care in an outpatient clinic setting over a fifteen-year period at the University of Minnesota (UMN) Masonic Children’s Hospital Pediatric Infectious Diseases (PID) Clinic. A retrospective review of clinic appointments identified 253 unique patients specifically evaluated over this time period for consideration of CMV infection. Of these, 242 were pediatric patients. The majority of the pediatric patients evaluated in the PID clinic were referred for either confirmed or suspected cCMV infection, including children referred for consideration of CMV as a potential reason for a failed newborn hearing screen (NHS) and/or for evaluation of CMV as a possible etiology for documented hearing loss. In total, 116 of the children evaluated during this time period (48%) were unequivocally confirmed as having cCMV infection, with an additional 37 (15%) presenting with presumed, probable, or possible cCMV infection. A total of 16 (7%) of the pediatric CMV cases were confirmed to be post-natally acquired infections. Of the 253 total patients, 11 (4%) of the referrals were for pregnant patients seeking advice about potential therapies in the setting of a known or suspected primary maternal infection during their pregnancies, with an attendant risk of fetal CMV infection. This overview of the demographics and referral patterns for patients evaluated for known or suspected CMV infections in a tertiary care center outpatient PID clinic will serve as a useful baseline assessment, even as future patterns of outpatient care are highly likely to evolve. We predict that PID clinic referrals for newborns identified by universal cCMV screening programs will result in a shift of the CMV outpatient population to healthier infants with clinically inapparent infections, and care will need to be taken by practitioners not to over-medicalize management for these asymptomatic newborns.

## 1. Introduction

Cytomegalovirus (CMV) infections, characterized by Weller as “ubiquitous infections with protean clinical manifestations” a generation ago [1,2], are commonly encountered and managed by pediatric infectious diseases (PID) clinicians in a variety of inpatient and outpatient care settings. These include the management of CMV infections in immunocompromised (including HIV-infected) children [3], infections in premature infants in the newborn intensive care unit (NICU) setting [4,5], and congenital cytomegalovirus (cCMV) infection [6,7]. Among all congenital and perinatal viral infections in the developed world (and probably the developing world, where it is less well studied [8,9]), cCMV is the most important cause of long-term disability in children. In addition to its importance as a cause of neurodevelopmental disability, cCMV is a major cause of sensorineural hearing loss (SNHL) in children [10,11,12]. Indeed, in the United States (US), cCMV causes more disability in children than the better-known entities of neural tube defects, fetal alcohol syndrome, congenital toxoplasmosis, HIV infection, and Down syndrome [13,14].

Given its impact on pediatric infectious disease practice, PID clinicians are often consulted to manage many aspects of CMV infection, including diagnostics, antiviral prophylaxis and therapy, risk assessment for immunocompromised patients as well as pregnant patients, and coordination of the monitoring of audiologic and neurodevelopmental outcomes. However, there are few reports in the literature that have attempted to describe the overall impact of cCMV on clinical practice in either the inpatient or outpatient practice settings. We therefore set out to describe the breadth and scope of pediatric outpatient clinic practice attributable to CMV in our PID clinic at the University of Minnesota (UMN). We predicted that the patterns of CMV-associated consultation and care would likely vary across pediatric sub-specialties and would reflect the underlying health issues in a given patient population (for example, pediatric academic medical centers with solid organ (SOT) and hematopoietic stem cell (HSCT) transplant programs would manifest different patterns of CMV-associated outpatient referral care than programs predominately specializing in maternal–fetal medicine, or in general pediatric care). Notably, we could find no published descriptions of the demographics and patterns of outpatient clinic care in which CMV infection was the major stated reason for a PID clinic referral and/or appointment. Published reports on how practitioners utilize PID consultations generally have focused on the inpatient care setting, and have included information about the general reasons for consultation [15,16], or presented metrics regarding the added value that an infectious diseases provider can provide for the management of specific diagnoses [17] or for specific categories of patients (for example, oncology patients [18]). Relatively little attention has been given in the literature to describing the breadth and scope of PID outpatient clinic referrals for any single pathogen, such as CMV. One reference provided an algorithm for how consultative referrals might be approached in the setting of known cCMV infection [19], but this paper referenced this in the context of subspecialty patient referrals that could improve the long-term management of children with symptomatic cCMV disease in need of longitudinal, multidisciplinary care. To our knowledge, no studies have enumerated and classified the impact of CMV more broadly on the scope and breadth of outpatient PID clinic referrals or clinical practice.

In this analysis, we assessed the impact of CMV infection on patterns of PID outpatient consultations over a 15-year period (2005–2020) at the University of Minnesota Masonic Children’s Hospital outpatient clinic. Our goal was to describe, in a single academic center, the impact of CMV over this time period on outpatient clinic referrals. We discovered, upon review of our experience, that outpatient clinic practice related to CMV was primarily focused on issues related to suspected or proven cases of cCMV. In recent years, knowledge and awareness of the impact of cCMV on pediatric practice have increased, driven in part by advocacy efforts from parent-based organizations [20,21,22] and by improved antenatal educational programs [23]. We suggest that referrals to PID clinicians for cCMV-related diagnosis and management questions are now likely to substantially increase, driven by the adoption by some states in the US of universal newborn cCMV screening programs [24,25,26]. Enhanced awareness of the importance of the association of cCMV with SNHL will also drive an increased number of PID clinic referrals [19]. In addition, increasing recognition of pediatric primary and secondary immunodeficiencies, where CMV is often an important opportunistic pathogen [27], will likely also contribute to an increase in future CMV-related PID referrals. Thus, we were interested in establishing baseline metrics for the impact of CMV on PID outpatient referrals during the time period covered by this retrospective chart review, with the goal of comparing how these practices may evolve in the future, now that universal cCMV screening programs have begun to be implemented in clinical practice, both in several Canadian provinces as well as in several states in the US.

## 2. Materials and Methods

### 2.1. Study Setting

MHealth Fairview/University of Minnesota Medical Center (UMMC) is a 1700-bed non-profit, tertiary, research and academic medical center located in Minneapolis, Minnesota. The Masonic Children’s Hospital is a 254-bed, freestanding, adjacent facility. It is affiliated with the UMN Medical School. The hospital provides comprehensive pediatric specialty and subspecialty care, including outpatient clinic care, to pediatric patients aged 0–21. The primary objective of this retrospective study was to describe the reason why a patient would present to the PID outpatient clinic at UMMC for evaluation of a documented or suspected CMV infection. The other goal was to provide a broad and descriptive examination of available CMV-related medical health information for patients attending this clinic. To accomplish these goals, we undertook a chart review of medical records generated during the course of care for patients seen in the PID outpatient clinic over a 15-year period (spanning 2005 through 2020) who were referred to the clinic specifically for an evaluation of CMV infection.

### 2.2. Patient Groups

This study was approved by the University of Minnesota Institutional Review Board (IRB) prior to reviewing data (IRB study identification number, STUDY00021015: Retrospective Review of Outpatient Clinic Visits for CMV). Inclusion criteria included patients of any age who were referred to and/or seen by the corresponding author’s care team (including pediatric infectious diseases fellows, pediatric residents, and medical students) over this time period. Patient encounters were coded as an evaluation or consultation/referral for evaluation for CMV infection. For each patient, corresponding health records were utilized to gather information pertaining to CMV infection. Patient charts included a mixture of AllScripts and EPIC as the source of the EMR data. The records were queried for demographic information, primary encounter dates, CMV-related symptoms, laboratory assays, imaging studies, and CMV-related diagnoses. Treatment and diagnostic criteria were abstracted from the patient encounter notes and from other linked records (audiology reports, laboratory assessments) available through the EMR. Some patients were referred for PID consultation, which was performed in parallel with a multidisciplinary clinical evaluation for SNHL-related concerns that was undertaken in the MHealth Lions Children’s Audiology and ENT Clinic. Some patients were referred by community primary care physicians because of an abnormal newborn screening test indicating the possibility of cCMV infection, and/or for evaluation following a “refer” on the newborn hearing screen (NHS). Key vocabulary words, phrases, and acronyms were used to search the medical records for CMV-related information. Keywords that were used included cCMV, CMV, ganciclovir, valganciclovir, dried blood spot (DBS), newborn hearing screening (NHS), head circumference, microcephaly, petechiae, intrauterine growth restriction (IUGR), retinitis, developmental delay, cerebral palsy, genetic test, small for gestational age (SGA), low birth weight (LBW), seizure, epilepsy, attention deficit/hyperactivity disorder (ADHD), hearing loss, sensorineural hearing loss (SNHL), cochlear implant, or hearing aids.

Laboratory records from diagnostic studies obtained at the time of the initial evaluation were recorded, including results of CMV urine and CMV plasma DNA quantification by PCR, CMV IgM antibodies, and CMV IgG. In some cases, clinic notes were linked to results from a CLIA-certified (24D1049829) laboratory that was utilized to test a patient’s newborn DBS, toward the goal of clarifying whether a referred patient carried a diagnosis of cCMV. Laboratory assays were used to help determine if CMV infections were congenital or acquired after birth, and were interpreted in the context of the maternal and child history, the physical examination, and (as available) ancillary imaging, audiologic, and ophthalmologic data. For cCMV infections, an algorithm was developed to assign the level of certainty for congenital infection (described below in Section 3) since the likelihood of vertical transmission could not always be ascertained with certainty.

### 2.3. Statistical Analyses

Descriptive statistics were performed using Prism 8.0 for MacIntosh (GraphPad Software, San Diego, CA, USA). Data were summarized as percentages and proportions for categorical measures. Numeric measures were summarized by calculations of means, medians, and standard deviations. *p*-values less than 0.05 were deemed statistically significant.

## 3. Results

### 3.1. Demographics

Our study identified 253 patients, which included 11 pregnant women who were evaluated because of concern regarding known or suspected primary maternal and/or fetal CMV infection, and 242 pediatric patients who were referred for evaluation of CMV infection, including infants with known or suspected cCMV. The characteristics of patients referred for consultation in the PID clinic are summarized in Table 1. Of note, there were four sets of twins and four individual twins (the womb mate was not included) that were evaluated as a part of this retrospective chart review.

Demographic information was obtained from the EMR and was collected based on patient responses. Patients included in this study were currently residing in the United States. A total of 248 patients (98.0%) were from the Midwest, with a majority residing in Minnesota. In total, 84.2% of patients identified English as their primary language.

Of the 242 pediatric patients, 129 were male and the other 113 were female, as indicated in the EMR (Table 1). The average age of the pediatric patients at first evaluation was 447 days of age (1.23 years). The youngest patient was 3 days old, and the oldest pediatric patient was 12 years old (4497 days). The average age of patients seen for referrals in the PID clinic and referred with a known diagnosis of cCMV was 0.8 years (294 days). For children referred with a concern for possible cCMV because of known SNHL and/or “refer” status on the NHS, the average age was 1.97 years (719 days). Of patients referred because of a positive newborn cCMV screening result, the average age at the time of initial consultation was younger, at 0.07 years (24.6 days).

### 3.2. Reason for Referral

**Figure 1:**
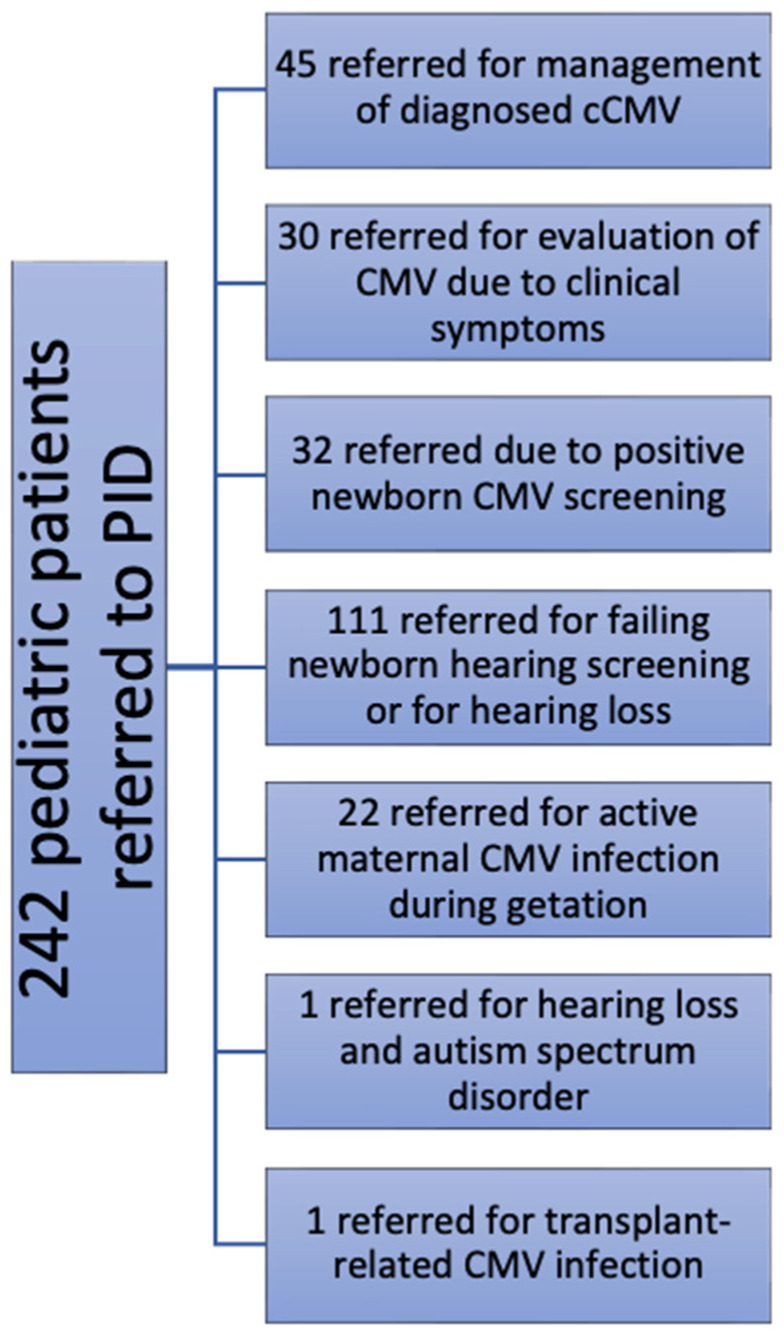
Flow chart for CMV pediatric referrals (*n* = 242). The number 111 represents the number of patients who were sent to the PID clinic for evaluation of known or suspected CMV infection specifically because of the hearing loss. The additional patients were referred to PID for other reasons as outlined in the text.

Patients were assigned to categories that we generated based on why they presented to the PID clinic for evaluation of possible CMV infection. Categories were as follows: evaluation for previously diagnosed cCMV (*n* = 45), referral for evaluation of possible cCMV (*n* = 30), identified by newborn screening for cCMV (*n* = 32), referred by audiology and/or otolaryngology practitioners (*n* = 111) because of concerns regarding hearing loss (SNHL 60, conductive 1, undifferentiated 50), concerns regarding hearing loss and autism spectrum disorder (*n* = 1), a documented history of a maternal CMV infection during pregnancy (*n* = 22), and transplant-related CMV infection (1; total, 242). The reasons for these PID clinic referrals (for pediatric patients) are summarized in the flow chart shown in Figure 1. Additionally, 11 pregnant patients (adult patients) were referred to the UMMC PID clinic for clinical evaluation and to assess for recommendations for possible maternal antiviral therapies (such as cytomegalovirus-immune globulin). These consultations were undertaken because of clinical suspicion for, and/or proven laboratory evidence of, maternal CMV infection during pregnancy (with or without proven fetal CMV infection). Although these were adult patients, since they were referred to the PID clinic, they were included in this analysis.

### 3.3. Patient Clinical Categories

**Figure 2:**
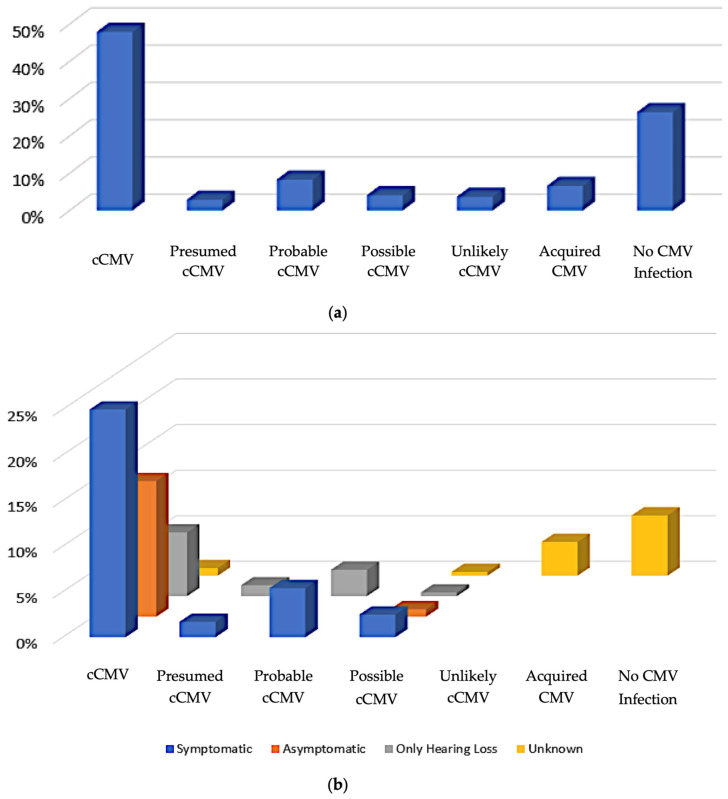
Schematic representation of likelihood of cCMV infection and corresponding disease categories for CMV patients evaluated in UMMC PID clinic, 2005–2020. (**a**) Percentage of pediatric patients differentiated into categories of likelihood of cCMV infection. (**b**) Percentage of pediatric patients assigned into categories of likelihood of cCMV infection, and as a function of symptomatic disease and/or SNHL [29], additionally segregated based upon their presenting signs/symptoms/clinical characteristics (*n* = 242).

As noted above, of the 253 patients whose medical records were evaluated, 242 were pediatric patients, and 11 were pregnant patients. Since there was a major emphasis in the PID clinic on management specifically of cCMV, we next tried to categorize the pediatric patients into groups using clinical information and laboratory data based upon the likelihood of cCMV infection. In our algorithm, we defined the patients who had positive CMV laboratory testing within the first 14 days of life and/or positive DBS testing [28] as being unequivocally classified as cases of cCMV infection (*confirmed cCMV*, Figure 2); this sub-group accounted for 48% (116/242) of all pediatric patients. Those with signs and symptoms suggestive of cCMV noted shortly after birth, and who had CMV-positive assays (but outside of the 14-day window) were presumed to have cCMV (*presumed cCMV*, Figure 2). Patients without definitive signs or symptoms of cCMV disease, and who had positive CMV testing outside the 14-day window but a positive test within the first month of life, were considered to have probably had cCMV infection (*probable cCMV*, Figure 2). Patients with signs and symptoms consistent with cCMV infections at birth but who did not undergo testing until after a few months of age, and for whom there were no other explanations for their symptoms, were assigned to a *possible cCMV* category. Pediatric patients with other diagnoses that accounted for their symptoms and who had a positive CMV test (but one that was obtained outside of the first month of life) were considered to be *unlikely cCMV* (Figure 2) since there were alternative explanations available to explain disabilities such as SNHL, developmental delay, etc. *Acquired CMV* was determined to be the patient’s category of classification if there was a documented negative CMV test at birth and a later positive CMV result. Patients who were CMV-negative by laboratory evaluation with alternative explanations for symptoms were deemed to not have CMV (*no CMV infection*, Figure 2). Disease categories as a function of the presence or absence of CMV-related symptomatic disease, as defined by Rawlinson et al. [29], are indicated in Figure 2b. Since CMV-related disease and/or SNHL could not be defined for acquired infection or for referred infants who ended up having no evidence of CMV infection, these are plotted as “unknown”.

The CMV infection status of the 253 patients is further summarized in Table 2. Specifically, the table indicates how many patients of those referred for evaluation had confirmation of any CMV infection.

### 3.4. Patient Characteristics

Of those pediatric patients evaluated for whom a full perinatal history was available, 140 were born at full term (37–42 weeks) and 48 were born at less than 37 weeks estimated gestational age (EGA; Table 1). Signs and symptoms suggestive of cCMV were evaluated. Of the 242 pediatric patients, 36 charts included diagnoses of intrauterine growth restriction (IUGR), and 46 infants were noted to have had a history of low birth weight/small for gestational age (LBW/SGA). Sixty-three medical charts specifically documented results with respect to the presence or absence of microcephaly; twenty-eight patients were listed as microcephalic and thirty-four were specifically noted to be normocephalic; and one patient was characterized as “plagiocephaly, not frankly microcephalic”. The average head circumference at birth for the microcephalic patients was 30.4 cm (±SD 1.6 cm; Table 3). The average newborn head circumferences for full term and premature infants were 34 cm and 30.7 cm, respectively. There were 87 patient encounters for whom newborn nursery histories were available; 12 of these reported the presence of neonatal petechiae, while the remaining 75 were noted to have normal skin with no rash. It was noted that four cCMV patients were born with sacral dimples. Patient disease categories are noted in greater detail in Table 3.

Of our 242 pediatric patients, there were 131 patients with available EMR records commenting on their developmental progress. Of those patients whose developmental staging was available for review, 60% (*n* = 78) were diagnosed with developmental delay or gross motor delay. Of the 131 patients with medical records addressing their development, 65 were confirmed to have cCMV. Of these children, 33 out of the 65 were diagnosed with developmental delay, while 32 of the 65 were deemed to be developing at a normal pace. Eighteen patients had both developmental delay and cerebral palsy and 13 of those 18 patients were confirmed or presumed to have cCMV.

Notably, not all patients summarized in Table 3 ended up having a diagnosis of cCMV. However, the table does underscore the types of clinical problems—hearing loss, developmental delay, white matter changes on MRI scan—that might lead to a referral for evaluation for possible cCMV infection. We noted that a number of patients seen in the PID outpatient clinic also carried the diagnosis of seizure disorder (34 patients). Two patients were specifically noted to have “absence” seizures. One patient was diagnosed with Lennox–Gastaut syndrome (with a documented 1p36 microdeletion), and one was found to have benign Rolandic epilepsy. Two additional patients were listed in their medical records as possibly having epilepsy, and one was found to have hippocampal abnormalities on brain imaging consistent with possible epilepsy. The reasons for referral for evaluation for CMV in the PID clinic were related to diagnostic uncertainty at the time of initial work-up, and cCMV was being considered in the differential diagnosis.

### 3.5. Laboratory Findings

Laboratory assays ordered at or soon after each patient’s primary evaluation were included in this study. For patients with an established diagnosis of cCMV referred to the PID clinic for evaluation, no additional virologic testing was performed. CMV urine PCR assays were obtained for 168 of the pediatric patients; 122 had positive results and 46 were negative. CMV whole blood and/or plasma assays were run on 128 patients. Not all patients underwent both CMV urine and blood or plasma PCR. Of the 60 who underwent both assays, 40 (67%) were positive for both, and 0 (0%) had positive CMV blood/plasma PCRs while also having negative urine PCRs. Eleven (18%) patients were positive for CMV in the urine and negative for CMV in the blood/plasma. CMV IgG was assessed on 96 patients, resulting in 77 positives, 18 negatives, and a single equivocal result. CMV IgM was determined for 100 patients, and 11 returned positive, suggesting that most patients were no longer in the acute stage of infection by the time they were evaluated in the PID clinic.

DBS testing for CMV was performed for 88 PID clinic patients as a component of their evaluation to retrospectively assess for (and, in some cases, attempt to confirm) cCMV infection, as described previously [28]. Out of those tested, 43 were positive. There were 28 medical records that also included the viral load data for the positive DBS specimens. The viral load mean among these positive DBS tests was 2.5 × 10^4^ copies/µg of genomic DNA.

### 3.6. Hearing Loss and Evaluation for Additional Non-CMV Etiologies

Within the medical record, 205 pediatric patients had the results of their NHS test recorded. At the time of initial consultation, 60% of the pediatric patients had been diagnosed with some form of hearing loss. Of those 145 patients with hearing loss, 81 (56%) had SNHL, 15 (10%) had conductive hearing loss, and 49 (34%) were designated as unspecified hearing loss. One hundred and nine patients utilized hearing aids, cochlear implants, or a combination of the two. Although these patients were referred for evaluation for possible CMV infection, some children had additional evaluations for other etiologies. Of the 145 pediatric patients identified with hearing loss according to the medical record, 54 were documented as having also undergone other testing to identify genetic etiologies for SNHL. One patient was treated for hearing loss related to tuberculosis infection. Usher’s syndrome was identified in three patients who were initially tested for cCMV, while two patients had dysmorphology suggestive of CHARGE syndrome.

Not all of the medical records indicated what specific genetic assays were performed. A single patient had their whole genome sequenced, and no pathologic variants associated with hearing loss were found. Two patients were found to have a pathogenic mutation in the *SLC6A4* gene and were diagnosed with Pendred syndrome as the cause of their hearing loss. Interestingly, a single patient formally diagnosed with symptomatic cCMV was also found to have a *COL4A4* gene mutation associated with Alport syndrome. For this patient, it was difficult to pinpoint which, if not both, factors contributed to the patient’s SNHL. Multiple other gene abnormalities associated with SNHL were noted, including TMC1, SHOX, and Connexin 26 gene variations (Table 4), as well as multiple chromosomal variations of unknown significance. Of note, one patient underwent genetic testing and was diagnosed with cystic fibrosis. One patient with hearing loss was referred for evaluation for possible cCMV, but was subsequently diagnosed with Down syndrome.

### 3.7. Treatment

Ganciclovir and valganciclovir are antiviral medications commonly used for the treatment of CMV infections. Patients identified in this retrospective chart review underwent various durations of treatment, the most common of which being oral valganciclovir therapy for six months. More than 60% of the pediatric patients in this series who were confirmed to have cCMV were treated with valganciclovir for six months. Other medication regimens included ganciclovir alone for a total of 6 weeks and combinations of intravenous ganciclovir, followed by oral valganciclovir, for various periods of time. The shortest duration of treatment for these patients was valganciclovir for two weeks, which was stopped due to side effects, and the longest was valganciclovir for a duration of one year. Notably, the parents and/or guardians of several patients diagnosed with cCMV declined the recommendation of antiviral therapy. Upon review of forty-four of the medical charts of patients with cCMV, no notation in the medical record was provided with respect to the question of whether antiviral therapy had been discussed and/or offered to these families in the UMN clinic; some of these children were on antiviral therapy that had already been commenced by the referring provider(s).

### 3.8. Ophthalmologic Findings

Ophthalmology evaluations are an important part of the diagnostic evaluation for cCMV [29]. CMV retinitis was noted in two patients in this retrospective review, one of which had confirmed cCMV. A total of 24% of those with accessible ophthalmic exams were noted to have various ophthalmologic findings, including three children with retinal scars; two with retinal pigment epithelium changes, consistent with CMV infection; two with evidence of retinal hemorrhage; one with congenital nuclear cataracts; and three with amblyopia (Table 3).

### 3.9. Neuroimaging Findings

Various forms of neuroimaging were performed on the patients in this retrospective chart review. Imaging included computerized tomography (CT) scans and X-rays of the skull and head, magnetic resonance angiography (MRA), and magnetic resonance imaging (MRI) of the brain, as well as cranial ultrasound (Table 3). White matter disease in the form of T2 hyperintensity and/or volume loss was noted on MRI in 32 patients, 20 of whom were confirmed to have cCMV.

### 3.10. Maternal Infections

Eleven women ranging from 20 to 41 years old were found to have primary CMV infections during pregnancy and were referred to the PID clinic for counseling regarding the potential utility of antiviral therapy. Of those women, four had been diagnosed with a first-trimester CMV infection. Three were diagnosed while in their second trimester, and a single patient was not diagnosed with an active CMV infection until their third trimester. It was not determined when the other three women were infected with CMV. Six of the eleven infants born to these women were subsequently evaluated in the PID clinic and were included in this analysis. Five of these patients were found to have cCMV (three symptomatic and two asymptomatic), while for one pregnant patient, it was determined that the viral infection did not transfer vertically to her newborn infant. Of those infants, four were treated with valganciclovir for a six-month duration.

## 4. Discussion

In this paper, we review our experience diagnosing and treating patients with cCMV and CMV infections over a 15-year span. We compiled diagnostic information and patient characteristics for all patients with proven or suspected CMV infection, including those that were seen with the intent of exploring and/or confirming a cCMV diagnosis.

Patients predominantly presented for evaluation of, and/or confirmation of, cCMV. The average age at which pediatric patients presented for their initial appointment in the PID clinic was 1.2 years of age. IUGR and SGA/low birth were the most recognizable features recorded in the EMR on the patients’ dates of birth. Other symptoms reported include petechiae and microcephaly. Less than half of patients had brain imaging performed prior to PID evaluation.

The most common reason for referral to the PID clinic was for evaluation for possible cCMV infection, either as an established diagnosis from an outside health care facility (*n* = 45), a suspected diagnosis based on clinical findings (*n* = 30), identification from newborn screening studies (*n* = 32), or concern for the possibility of vertical transmission in the setting of known symptomatic maternal infection during pregnancy (*n* = 22). The second most common reason for referral for CMV evaluation was for the evaluation of unexplained SNHL. Patients were referred by audiology and/or otolaryngology practitioners due to documented hearing loss (SNHL, sixty; conductive, one; undifferentiated, fifty) or for a failed (“refer” status) NHS, which in any case typically prompted a diagnostic evaluation that included testing for cCMV.

Surprisingly, we noted only one PID clinic referral that was specifically for the management of CMV disease in a transplant patient. This patient was an 11-year-old male who underwent a living paternal donor kidney transplant and subsequent immunosuppression on tacrolimus and mycophenolate mofetil. He was hospitalized for right-sided middle-lobe polymicrobial pneumonia, was positive for CMV and *Mycoplasma pneumoniae*, and was subsequently treated with 10 days of azithromycin and 14 days of ganciclovir. Approximately one year later, he was again hospitalized for CMV, adenovirus type C, *Haemophilus influenzae*, and *Actinomyces* pneumonia. This time he was discharged with oral valganciclovir for 6 weeks, and subsequently presented to PID clinic for management of CMV infection and for an immunodeficiency evaluation. We attribute this low number of specific referrals for management of CMV infections in transplant/oncology patients to the fact that the UMMC pediatric hematology/oncology and transplantation services have well-developed management algorithms for management of CMV infections post-transplantation, and typically, referral to PID clinic is not required specifically for CMV-related issues in our institution. In addition, although CMV infection is a common management concern in transplant patients, it is just one of a myriad of infectious disease-related issues in this patient population, and this may explain why specific referrals for CMV were rare, since other infection issues may have generated more pressing concerns for these patients.

In summary, of our 242 pediatric patients evaluated over the past 15 years, the majority of outpatient clinical evaluations in our institution were focused on confirmed, probable, or suspected cases of cCMV infection. In total, 116 were unequivocally confirmed to have cCMV infection, and an additional 37 had either possible, probable, or presumed cCMV based on their overall clinical and laboratory assessments. The laboratory, imaging, and general clinical characteristics of these patient groups are summarized in this report. As a referral center, we tended to evaluate more complex cases of suspected cCMV infection with a range of attendant disabilities (SNHL, neurodevelopmental disorders, seizure disorders) and infants born to pregnant persons with known or suspected maternal-fetal transmission during pregnancy. A majority of our cCMV patients were treated with valganciclovir for six months. Other medication regimens included ganciclovir for 6 weeks and a combination of ganciclovir and valganciclovir for various periods of time ranging from two weeks to one year of medical therapy.

There was a high frequency of known or suspected SNHL cases among our CMV referrals; many children with SNHL (as well as other concerns prompting referral) did not, in fact, end up having any evidence of CMV infection. Hearing loss was a pervasive concern. In total, of the 242 pediatric referrals for evaluation for CMV infection, we found through a chart review that 152 had some degree of hearing loss, regardless of whether NHS had been performed or what the results demonstrated. The chart review also indicated that 113 patients in this group of 242 patients had NHS screening data available for our review (many of these overlapping with the 152 patients with documented hearing loss), demonstrating that they had referred (failed) on their NHS for at least one ear. Patients in this group were referred for further hearing evaluation and to the PID clinic for evaluation for possible CMV infection. As noted, there is some crossover between these 113 and 152 patients; we did not have access to all of their NHS and audiologic tests, so it was impossible to fully differentiate these two groups.

Of those pediatric patients with audiologic confirmation of hearing loss, 88/152 were categorized into either the category, as defined in Section 3.3, of “proven cCMV” (*n* = 64) or the categories of “presumed or probable” cCMV (*n* = 24). Thus, overall, there was a strong association between a PID clinic referral and hearing loss to evaluate for possible or proven CMV infection in our institution. This is likely explained by the fact that our group has collaborated closely for many years with the Lions Children’s Audiology and ENT Clinic at the UMN [30], resulting in a large number of referrals to rule out cCMV in the context of both documented SNHL and in the context of “refer” status on the newborn hearing screen (NHS). Only a small percentage of infants who “refer” to the NHS will, in fact, have either demonstrable SNHL and/or evidence of cCMV infection [31].

One observation we offer from this analysis is that it is not necessary to refer an infant to the PID clinic only because of a “refer” status on NHS, without at least some documentation of CMV infection being present, either through virologic testing or through the finding of signs/symptoms associated with cCMV. To avoid unnecessary PID clinic referrals and attendant potential parental anxiety, a “targeted” screening approach [32], whereby a specimen (saliva or urine) is sent for CMV testing in infants that “refer” on the NHS, may be a more reasonable, efficient, and cost-effective approach than a formal clinic referral, since the diagnosis of cCMV can easily and quickly be excluded (in most cases) by obtaining a PCR test of saliva or urine for the newborn infant prior to discharge from the newborn nursery.

Given the rising adoption of universal newborn cCMV screening programs by various states in the US, we expect the rate of referrals to PID physicians for diagnosis as well as long-term management to rise. Our description of referral patterns and patient characteristics may prove useful when identifying which patients are more or less likely to be referred for further evaluation of possible cCMV. Currently, the bias of pediatric infectious diseases clinicians is focused on the management of symptomatic cCMV disease—because, after all, these are the cases that are recognized clinically. Such “symptomatic” cases are unusual (most cases of cCMV are asymptomatic), but they are important to recognize since there is a benefit to be gained in many infants through the use of long-term valganciclovir therapy [33]. As universal cCMV screening moves forward, care must be taken not to “over-medicalize” cCMV [34]. It is imperative that all children with cCMV undergo frequent audiologic monitoring, given the risk for SNHL that is present even in children who pass their NHS. Caution is recommended, however, with respect to the risk of over-prescribing agents such as valganciclovir [35] in children with asymptomatic cCMV. The data reported in this fifteen-year retrospective review can serve as a metric for the patterns of referral observed in an academic pediatric medical center from the pre-universal screening era. Prospective controlled studies examining neurodevelopmental outcomes in infants with cCMV, who have heretofore been considered to have a normal prognosis for cognitive development, are needed to better ascertain if there are any subtle phenotypes that have escaped clinical detection in the pre-screening era.

## 5. Conclusions

CMV infections have a substantial impact on outpatient clinic practice for PID physicians. Patients referred to PID for evaluation of potential cCMV infections have widely varying presentations ranging from asymptomatic to highly symptomatic. In this retrospective 15-year review in our academic medical center, we found that the major reasons for referral for evaluation of CMV infection centered around proven or suspected cCMV, with a particular emphasis on hearing loss. The average age of presentation at the PID clinic was 1.2 years of age, making definitive diagnosis difficult if testing had not been performed in the first weeks of life, since post-natal acquisition of CMV is common and cCMV is hard to prove (outside of finding viral shedding in the newborn period). Minnesota initiated universal newborn screening for cCMV in 2023, and this will likely change the patient mix of CMV cases in PID clinics, with a substantial increase in asymptomatic infants. This retrospective overview of pediatric CMV infections in a PID outpatient clinic will serve as a valuable reference point for understanding potential shifts in referral patterns as universal cCMV screening becomes more prevalent in clinical practice. PID clinic referrals for newborns identified by universal cCMV screening programs will result in many asymptomatic infants, without clinically apparent cCMV infections, presenting for PID consultation. Practitioners should take care not to over-medicalize the management of these well-appearing newborns, and in particular, to be cautious about the prescription of potentially toxic nucleoside antivirals that are of unproven benefit in the asymptomatic infant.

## Figures and Tables

**Table 1:** Demographic information on CMV patients evaluated in UMMC PID outpatient clinic.

Patient Characteristic	Result
Pediatric patients	242 (95.7%)
Maternal patients	11 (4.3%)
Mean pediatric age ^1^	1.2 years (3 days–12 years)
Mean maternal age ^1^	34 years (20–41 years)
Sex (pediatric patients)	Male 129 (53.3%)
	Female 113 (46.7%)
Race/Ethnicity	
White/Caucasian	170 (67.2%)
Hispanic/Latino	16 (6.3%)
Black or African American	28 (11.1%)
Asian, Native Hawaiian or Other Pacific Islander	0 (0.0%)
American Indian/Alaska Native	2 (0.8%)
Middle Eastern or North African	0 (0.0%)
Choose not to disclose	31 (12.3%)
Other	6 (2.4%)
Residence	
Minnesota	230 (90.9%)
Wisconsin	9 (3.6%)
North Dakota	4 (1.6%)
Iowa	3 (1.2%)
Georgia	2 (0.8%)
Michigan	1 (0.4%)
Alaska	1 (0.4%)
Virginia	1 (0.4%)
Illinois	1 (0.4%)
Arizona	1 (0.4%)
Primary Language	
English	213 (84.2%)
Spanish	12 (4.7%)
American Sign Language	8 (3.2%)
Somali	5 (2.0%)
Declined to list	15 (5.9%)
Gestation (pediatric patients)	
Full term (37–42 weeks)	140 (58.0%)
Premature (<37 weeks)	48 (20.0%)
Late term (>42 weeks)	1 (0.4%)
Data not available	53 (22.0%)

^1^ Age at time of initial consultation.

**Table 2:** Confirmation of CMV status in patients evaluated in PID clinic, 2005–2020 (*n* = 253).

Pediatric Patients	242
Proven Congenital CMV	116
Presumed cCMV	7
Probable cCMV	20
Possible cCMV	10
Unlikely cCMV	9
Acquired CMV	16
No CMV infection	64
Adult Patients	11
Proven CMV	11 (100%)
Total	253

**Table 3:** Clinical, laboratory, and imaging findings for all pediatric patients evaluated in the UMMC PID clinic for proven, suspected, or possible CMV infection from 2005–2020 (*n* = 242).

Characteristic	Finding	Number (%)
Clinical/Historical	Intrauterine growth restriction	36 (14.9%)
	Low birth weight/small for gestational age	46 (19.0%)
	Petechiae at birth	13 (5.4%)
	Developmental delay/gross motor delay	78 (32.2%)
	Cerebral palsy	18 (7.4%)
	Attention deficit/hyperactivity disorder	13 (5.4%)
	Seizures	34 (14.0%)
	Absence	2 (0.8%)
	Epilepsy	6 (2.5%)
	Microcephaly	28 (11.6%)
	Head circumference at birth; average	
	Full term infants	34.0 cm
	Premature infants	30.7 cm
	Microcephalic newborns	30.4 cm
	Newborn hearing screening (*n* = 205)	
	Passed bilaterally	110 (53.7%)
	Refer for repeat/additional testing	95 (46.3%)
	Hearing loss (*n* = 145)	
	SNHL	81 (55.9%)
	Conductive hearing loss	15 (10.3%)
	Unspecified hearing loss	49 (33.8%)
	Hearing assistance device used	109 (75.2%)
Lab Findings	CMV urine quantitative assays (*n* = 168)	
	Positive	122 (72.6%)
	Negative	46 (27.4%)
	CMV blood/plasma assays (*n* = 128)	
	Positive	67 (52.3%)
	Negative	39 (30.5%)
	Non-quantifiable (<137 copies/mL)	22 (17.2%)
	CMV IgM (*n* = 100)	
	Positive	11 (11.0%)
	Negative	86 (86.0%)
	Unequivocal	3 (3.0%)
	CMV IgG (*n* = 96)	
	Positive	77 (80.2%)
	Negative	18 (18.8%)
	Equivocal	1 (1.0%)
	Dried blood spot testing (*n* = 88)	
	Positive	43 (48.9%)
	Negative	25 (28.4%)
	Not reported	20 (22.7%)
	DBS CMV viral load (*n* = 28, data available)	
	Mean	2.3 × 10^4^ cp/μg
	Standard error of mean	8.1 × 10^4^ cp/μg
Ophthalmology	Ophthalmological exam in EMR (*n* = 88)	
	No abnormality reported	67 (76.1%)
	CMV retinitis	2 (2.3%)
	Retinal scar	3 (3.4%)
	Retinal dysfunction	1 (1.1%)
	Retinal hemorrhage, no retinitis	2 (2.3%)
	Retinopathy of prematurity	5 (5.7%)
	Retinitis pigmentosa	3 (3.4%)
	Optic atrophy	1 (1.1%)
	Congenital nuclear cataracts	1 (1.1%)
	Amblyopia	3 (3.4%)
Brain Imaging	Skull CT (*n* = 26)	
	Normal	15 (57.7%)
	Otomastoiditis	6 (23.1%)
	Dysplasia of cochlea	3 (11.5%)
	Dysplasia, semicircular (SC) canal	1 (3.8%)
	Dysplasia of cochlea and SC canal	1 (3.8%)
	Head CT (*n* = 16)	
	Normal	12 (75.0%)
	Inflammatory change of middle ear	1 (6.3%)
	White matter hypodensities	1 (6.3%)
	Enlarged vestibular aqueducts	2 (12.5%)
	Skull X-ray (*n* = 1)	
	Normal	1 (100%)
	MRA (*n* = 1)	
	Normal	1 (100%)
	MRI (*n* = 97)	
	Normal	14 (14.4%)
	White matter disease (T2 intensities)	32 (33.0%)
	Neuronal migration defect	1 (1.0%)
	Delayed myelination	6 (6.2%)
	Calcifications	5 (5.2%)
	Ventriculomegaly	3 (3.1%)
	Cortical malformations	4 (4.1%)
	Clumping of basal ganglia	1 (1.0%)
	Otomastoiditis	4 (4.1%)
	Dysplasia of cochlea	1 (1.0%)
	Hemorrhage	4 (4.1%)
	Sequelae of hypoxia/ischemia	2 (2.1%)
	Polymicrogyria	4 (4.1%)
	Lissencephaly	1 (1.0%)
	Periventricular cyst	5 (5.2%)
	Arachnoid cyst	2 (2.1%)
	Subependymal cyst	1 (1.0%)
	Leukodystrophy	2 (2.1%)
	Cerebral atrophy	1 (1.0%)
	Pachygyria	1 (1.0%)
	Microcephaly	2 (2.1%)
	Prominent subarachnoid	1 (1.0%)
	Head ultrasound (*n* = 82)	
	Normal	51 (62.2%)
	Echogenic foci	8 (9.8%)
	Mineralizing vasculopathy	8 (9.8%)
	Ventriculomegaly	1 (1.2%)
	Leukomalacia	1 (1.2%)
	Choroid plexus cyst	5 (6.1%)
	Periventricular cyst	1 (1.2%)
	Subependymal cyst	3 (3.7%)
	Hemorrhage/prior hemorrhage	4 (4.9%)

**Table 4:** Alternative diagnoses established in children with SNHL originally referred for evaluation for possible CMV infection.

Genetic Condition	Patients Identified
Usher syndrome	3
Pendred syndrome	2
Connexin 26 mutations	2
Monoallelic deletion of *SHOX* gene	1
Alagille syndrome	1
TMC1 DFNB7/11 mutation	1
COL4A4 (Alport syndrome) ^1^	1
CHARGE syndrome	2

^1^ This patient also had symptomatic cCMV infection.

## Data Availability

The data presented in this study are available upon request from the corresponding author.
